# Lack of social support associated with major depressive disorder in middle-aged and older adults from the United States: A propensity score-matched cohort study

**DOI:** 10.1371/journal.pone.0340260

**Published:** 2026-01-16

**Authors:** Ping Li, Kunlun Wu

**Affiliations:** 1 Department of Neurosurgery, Ankang Central Hospital, Ankang City, Shaanxi, China; 2 Department of Neurosurgery, Xi’an No.3 Hospital, The Affiliated Hospital of Northwest University, Xi’an, Shaanxi, China; University of Kansas School of Medicine Wichita, UNITED STATES OF AMERICA

## Abstract

**Background and objective:**

Current widely used depression assessment criteria may not accurately reflect the true prevalence of depression, and previous studies have insufficiently addressed the multidimensional nature of social support**.** Research on the relationship between depression and specific dimensions of social support remains limited, which impedes the targeted allocation of scarce social resources to mitigate the disease burden. This study aimed to explore the association between multi-dimensional social support and major depressive disorder (MDD) in the US population, using depression assessment criteria consistent with current epidemiological data.

**Method:**

We analyzed data from the National Health and Nutrition Examination Survey (NHANES). After propensity score matching (PSM), 123 participants with MDD (Patient Health Questionnaire-9 score ≥15) and 543 without MDD were included. Social support was assessed using five-dimensional questionnaires and each dimension was dichotomously scored (1 = presence of support, 0 = absence of support), yielding a total score ranging from 0 to 5. Participants were categorized into three social support groups: Low group (0–1), Middle group (2–3), and High group (4–5). Logistic regression models were used to examine the association between social support and MDD, and subgroup analyses were performed to assess consistency across populations.

**Results:**

Compared with participants without MDD, those with MDD had significantly lower proportions of adequate social support across all five dimensions. Binary logistic regression analyses showed that higher social support levels were associated with a reduced risk of MDD (Middle vs Low group: odds ratio [OR], 0.40; 95% CI, 0.22–0.73; P = 0.003; High vs Low group: OR, 0.15; 95% CI, 0.08–0.29; P < 0.001). Financial support exhibited the strongest inverse association with MDD, while adequate religious activity showed the weakest inverse association. The relationship between social support and MDD remained consistent across all subgroup analyses.

**Conclusions:**

In brief, sufficient social support, especially financial support, was linked to a significantly lower risk of MDD among the middle-aged and elderly population in the United States.

## 1. Introduction

Depression, affecting an estimated 350 million people worldwide, is one of the most prevalent and debilitating mental illnesses [1,2]. Major depressive disorder (MDD) significantly impacts patients’ quality of life [3], work, and social function [4,5], and is associated with cognitive disorders, cardiovascular disease, and various diseases [6–9]. MDD is increasingly becoming a major contributor to the global health-related and economic-related burden [4]. Especially in the United States, MDD is the largest burden of all mental and behavioral health disorders, accounting for 2.7 million disability-adjusted life years in 2016 [10], and the productivity loss due to MDD has increased from $236 billion in 2010 to $326 billion in 2018 [11].

Social support, a multifaceted concept encompassing emotional, instrumental, and informational assistance [12], plays a crucial role in both physical and mental health. It can be objectively measured through indicators like marital status, connections with relatives and friends, religious beliefs and group membership, as appropriate, and subjectively by the perception of help from others [13]. While existing literature highlights a robust association between social support and depression, several limitations persist. Some studies have oversimplified social support scales, focusing solely on emotional support while neglecting religious and instrumental aspects, potentially leading to an incomplete assessment of social support [13,14]. Some studies biased the definition of social support toward real-life participation while overlooking emotional aspects [15]. Additionally, many studies were conducted in specific population, such as the veterans [16], pregnant woman [17], or patients with cardiovascular disease [18,19]. The sample of most previous studies focus on overall mild and above depression population and did not distinguish depression severity. Several studies concentrated on MDD [20,21], but with the criteria that tended to overestimate MDD prevalence rate [22].

Considering the high incidence and recurrence rate as well as the huge social and economic burden of MDD in the United States and some limitations in the previous researches, it is necessary to further clarify the influence of multi-dimensional social support on MDD in middle-aged and elderly general population, which will help to lower the occurrence and development of MDD by improve social support from a specific dimension, and so as to specifically reduce the social burden on medical expenses. Thus, we employed data from the National Health and Nutrition Examination Survey (NHANES) to investigate the association between various types of social support and MDD.

## 2. Method

### 2.1. Study design and sample

**Fig 1:**
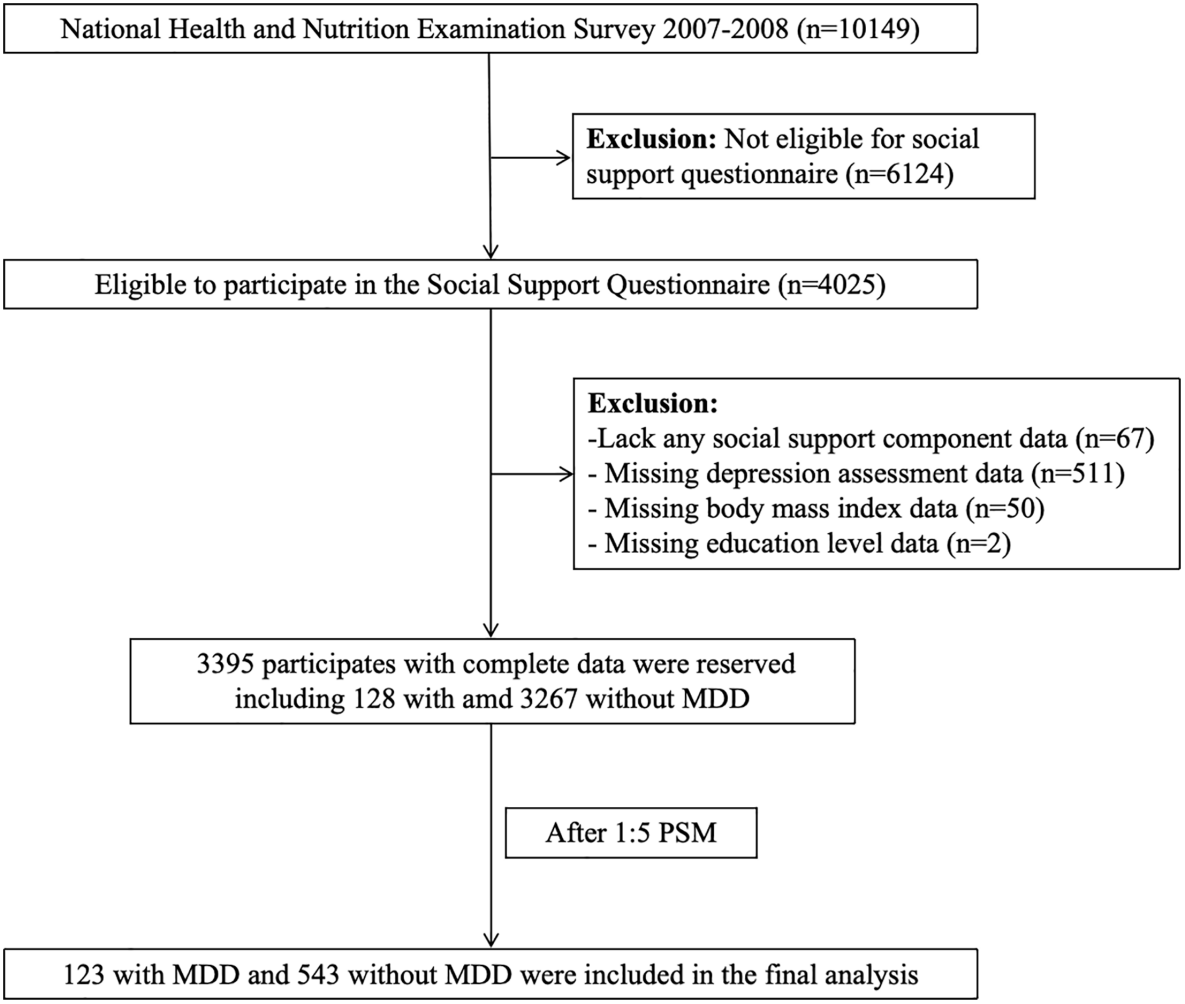
Study flowchart. Abbreviations: MDD, Major Depressive Disorder; PSM, Propensity score matching; NHANES, National Health and Nutrition Examination Survey.

NHANES is a cross-sectional survey employing a multi-stage, complex probability sampling design, which is specifically developed to monitor the health status of the U.S. population [23]. The present cross-sectional study utilized data from NHANES to explore the association between multidimensional social support and MDD among middle-aged and elderly adults in the United States. Demographic information, self-reports, and questionnaire data of all participants were obtained through household interview. Participants from the NHANES who completed the social support questionnaire between 2007–2008 were included. Notably, only individuals aged 40 years or older were eligible to complete this social support questionnaire (n = 4025). Participants were excluded if they had missing data on any item of the social support components (n = 67), the depression screening scale (n = 511), or other essential information (n = 52). After applying these eligibility criteria, a total of 3395 individuals were included in this study. Propensity Score Matching (PSM) was then performed, and the final analysis included 123 participants with and 543 without MDD. The detailed study flowchart is presented in Fig 1.

### 2.2. Ethical issues

All the participants were informed verbally about the aims, methods and procedures of the survey upon their recruitment and signed an informed consent voluntarily. The protocol of NHANES was approved by the National Center for Health Statistics. Detailed information on enrollment, procedures, and other NHANES data can be obtained by visiting https://www.cdc.gov/nchs/nhanes/index.htm.

### 2.3. Social support questionnaire

The social support questionnaire consisted of five dimensions which are emotional support, financial support, frequency of attending religious services, number of close friends, as well as marital status. The questions were selected from the Yale Health and Aging Study and the Social Network Index-Alameda County Study. Moreover, it’s structure and predictive effectiveness for NHANES have been proven in many previous studies [24–26]. The detailed questionnaire contents were as follows, emotional support is assessed by asking the question “Can you count on anyone to provide with emotional support such as talking over problems or helping make a difficult decision?” The question “Could you count on anyone to help, for example, by paying any bills, housing costs, hospital visits, or providing with food or clothes?” was used to assess the financial support. For the above two questions, the answers of “yes” were assigned as “1 point.” While those who answer “no”, “don’t need” or “don’t accept” got “0 point.” The status of being married or living as married was awarded 1 point and the status of widowed, divorced, separated, never married was representative 0 point. 1 point was assigned if attending at least four religious services per year (How often do you attend church or religious services?) or having four or more close friends (In general, how many close friends do you have?). For any question, the answer of “don’t know” or refusing to answer were considered as missing data and were excluded from our study. Therefore, the social support score ranged from 0 to 5 points.

### 2.4. MDD assessment

MDD was assessed by the Patient Health Questionnaire (PHQ-9), a version of the Prime-MD diagnostic instrument. They were a self-reported assessment of the past 2 weeks, based on nine DSM-IV signs and symptoms from depression. The nine symptom questions are scored from “0” (not at all) to “3” (nearly every day). Severity of depression can be defined by several cut points from the total score that ranges from 0−27 points. PHQ-9 scores within 0−4, 5−9, 10−14, 15−19, 20−27 are considered as having minimal, mild, moderate depressive, moderately severe, and severe depressive symptoms, respectively [27]. In the validity verification study of PHQ-9 by Kurt et al., scores of 10–14 only represented a spectrum of MDD patients and scores of 15 or greater usually indicated MDD. PHQ-9 scores ≥15 was proved to identify MDD with 95% specificity [27]. Although PHQ-9 score ≥10 had a sensitivity of 88% and a specificity of 88% for MDD, this 10-point cut-off value for MDD greatly exaggerated the prevalence rate and was inconsistent with the actual epidemiological data [22]. To minimize the impact of inclusion of participants without MDD on the final conclusions, we used a higher cut-off score of 15 points to identify participants with MDD [22,28].

All the questionnaires were asked by trained interviewers using the Computer Assisted Personal Interviewing (CAPI) system during the Mobile Examination Center private interview. The CAPI system was programmed with built-in consistency checks to reduce data entry errors, and approximately 5% of the interviews were recorded and reviewed for quality control purposes.

### 2.5. Other variables

Other variables included age, sex, education level, race, and Body mass index (BMI). Age was divided into five categories: 40–49, 50–59, 60–69, 70–79, and 80 years old or older. Education level was divided into four levels: less than high school graduate, high school graduate or equivalent, some college or associate of arts degree, and college graduate or above. BMI was calculated as weight divided by height. Race is described as non-Hispanic White; Mexican American and other Hispanic; non-Hispanic Black; Other.

### 2.6. Statistical analyses

Continuous variables were reported as means (standard error) and analyzed using Student’s t-test or Manne Whitney U-test. Categorical variables were summarized as and number (percentage) and chi-squared test or Fisher’s exact test were used for comparison.

Previous studies have demonstrated that age, sex, obesity, education level, and ethnicity exert significant influences on MDD to varying degrees [29–32]. In our study, propensity scores were calculated based on the following baseline covariates: age, sex, BMI, education level, and race. Collinearity testing was performed for all potential covariates, and no collinearity was detected among these variables. A nearest-neighbor matching strategy (1:5 PSM) with a caliper width of 0.1 was adopted to generate MDD cohort and non-MDD cohort. Balance between the two cohorts was assessed using the standardized mean difference (SMD), where an SMD value of less than 0.10 indicated adequate balance. According to the social support score level with 0–1, 2–3, 4–5 scores, respectively, corresponding to Low group, Middle group, and High group.

Subsequently, receiver operating characteristic (ROC) curves were employed to evaluate the discrimination ability of social support for MDD. Binary logistic regression analysis was conducted to calculate the odds ratio (OR) and 95% confidence interval (CI), thereby estimating the association between the three social support levels (Low group, Middle group, and High group) and MDD after PSM. Additionally, we assessed the association between each of the five individual social support components and MDD, aiming to further identify which specific aspect exerted the most significant impact on MDD.

To explore potential heterogeneity, subgroup analyses stratified by gender and age were additionally performed. Finally, sensitivity analyses were performed to confirm the robustness of the association between social support and MDD, with two alternative social support grouping strategies utilized: 1) dichotomization into two categories (0–2 points vs. 3–5 points) and 2) categorization as an ordinal variable into six distinct groups (0, 1, 2, 3, 4, 5). All statistical tests were two-tailed, with a significance level set at 0.05. These analyses were implemented using SPSS version 25 and R software version 4.4.2.

## 3. Results

### 3.1. Baseline characteristics

The baseline characteristics of all participants were described in Table 1. Age (P = 0.001), sex (P = 0.001), education level (P = 0.001), and race (P = 0.028) showed statistically significant differences between participants with and without MDD before PSM. While after PSM, all the baseline differences of age (P = 0.83), sex (P = 0.18), BMI (P = 0.55), education level (P = 0.70), and race (P = 0.87) were well balanced across the two groups. According to S1 Fig after matching, all the SMD of baseline variables were less than 0.1, also indicating that the baseline variables were well balanced. As shown in the Fig 1, a total of 543 participants in NHANES without MDD and 123 participants with MDD were included in our final analyses after PSM.

**Table 1:** Descriptive statistics of baseline characteristics among general adults aged 40 years and older from NHANES before and after PSM.

Variables	Total	Before PSM			Total	After PSM		
		Without MDD	MDD (PHQ-9 ≥ 15)	P		Without MDD	MDD	P
Participates, n	3395	3267	128		666	543	123	
Age, year				**0.001**				0.83
40-49		789 (24.2)	35 (27.3)			168 (30.9)	34 (27.6)	
50-59		753 (22.3)	46 (35.9)			162 (29.8)	42 (34.1)	
60-69		838 (25.7)	31 (24.2)			139 (25.6)	31(25.2)	
70-79		594 (18.2)	11 (8.6)			57 (10.5)	11(8.9)	
≥80		293 (9.0)	5 (3.9)			17 (3.1)	5 (4.1)	
Male, n (%)		1614 (49.4)	44 (34.4)	**0.001**		230 (42.4)	44 (35.8)	0.18
Body mass index, kg/m²		29.3 ± 6.4	30.2 ± 7.2	0.18		29.6 ± 6.8	30.1 ± 7.2	0.55
Education level, n (%)				**0.001**				0.70
Less than high school graduate		1024 (31.3)	57 (44.5)			206 (37.9)	53 (43.1)	
High school graduate/GED or equivalent		811 (24.8)	30 (23.4)			133 (24.5)	30 (24.4)	
Some college or AA degree		779 (23.8)	32 (25.0)			161 (29.7)	31 (25.2)	
College graduate or above		653 (20.0)	9 (7.0)			43 (7.9)	9 (7.3)	
Race, n (%)				**0.028**				0.87
Mexican American		490 (15)	18 (14.1)			74 (13.6)	18 (14.6)	
Non-Hispanic white		1675 (51.3)	51 (39.8)			247 (45.5)	51 (41.5)	
Non-Hispanic black		654 (20)	33 (25.8)			127 (23.4)	32 (26.0)	
Other race		448 (13.7)	26 (20.3)			95 (17.5)	22 (17.9)	

Abbreviations: NHANES, National Health and Nutrition Examination Survey; PSM, Propensity score matching; MDD, Major Depressive Disorder; PHQ-9, Patient Health Questionnaire; GED, General Education Development; AA, Associate of Arts. Variables with a p-value <0.05 are depicted in bold.

**Fig 2:**
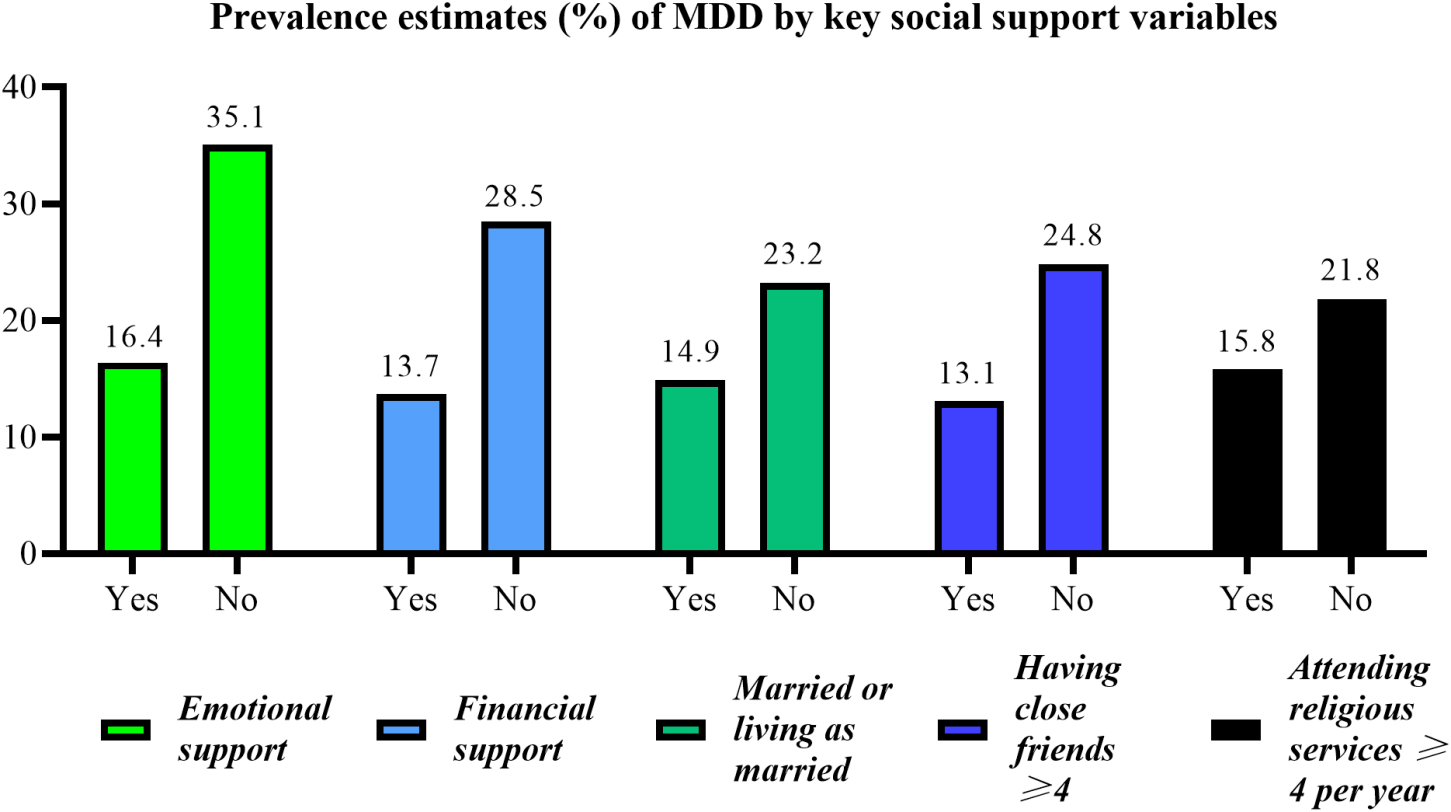
Prevalence estimates (%) of MDD stratified by the five components of social support questionnaire. Abbreviations: MDD, Major Depressive Disorder.

As shown in Fig 2, the prevalence of MDD was significantly lower in participants with partner support, emotional support, economic support, religious support, or intimate friend support compared to those without the corresponding support (14.9% vs 23.2%, 16.4% vs 35.1%, 13.7% vs 28.5%, 15.8% vs 21.8%, 13.1% vs 24.8%; All P values <0.05).

### 3.2. Social support and MDD

As shown in S1 Table, the incidence proportion of MDD in the low social support group was significantly higher than that in the high social support group (42.3% vs. 9.8%; P < 0.001). Area under the ROC curves (AUC) indicated that social support score had a certain discrimination for MDD (AUC = 0.67, 95% CI: 0.61–0.73, P = 0.029, S2 Fig).

Binary logistic regression analysis showed that compared with the low social support group, the high social support group had a significantly lower risk of MDD (OR = 0.15, 95% CI: 0.076–0.29, P < 0.001; Table 2). Additionally, the middle social support group also had a lower risk of MDD than the low social support group (OR = 0.40, 95% CI: 0.22–0.73, P = 0.003; Table 2).

**Table 2:** Binary logistic regression analysis of association between three social support groups and MDD after PSM.

Social support groups	OR (95% CI)	P
Low group (social support score = 0–1)	Reference	
Middle group (social support score = 2–3)	0.40 (0.22-0.73)	**0.003**
High group (social support score = 4–5)	0.15 (0.076-0.29)	**<0.001**

Abbreviations: MDD, Major Depressive Disorder; PSM, Propensity score matching; OR, Odds Ratio; CI: Confidence Interval. Variables with a p-value <0.05 are depicted in bold.

To further clarify which component of social support had the strongest correlation with MDD, additional analyses were conducted. As shown in Table 3, economic support exhibited the strongest negative correlation with MDD (β = −0.72, P = 0.001). The remaining components, ranked by the strength of their correlation with MDD (from strongest to weakest), were as follows: emotional support (β = −0.59, P = 0.038), having a sufficient number of intimate friends (β = −0.54, P = 0.038), marital status (β = −0.46, P = 0.028), and sufficient frequency of religious activity participation (β = −0.28, P = 0.18).

**Table 3:** Binary logistic regression analysis of association between all social support components and MDD after PSM.

Social support components	β	OR (95% CI)	P
Emotional support	−0.59	0.55 (0.32-0.97)	**0.038**
Financial support	−0.72	0.49 (0.32-0.75)	**0.001**
Marital status	−0.46	0.63 (0.42-0.95)	**0.028**
No. of close friends	−0.54	0.58 (0.38-0.89)	**0.012**
Annual religious services attendance	−0.28	0.76 (0.51-1.14)	0.18

Abbreviations: MDD, Major Depressive Disorder; PSM, Propensity score matching; OR, Odds Ratio; CI: Confidence Interval. Variables with a p-value <0.05 are depicted in bold.

**Fig 3:**
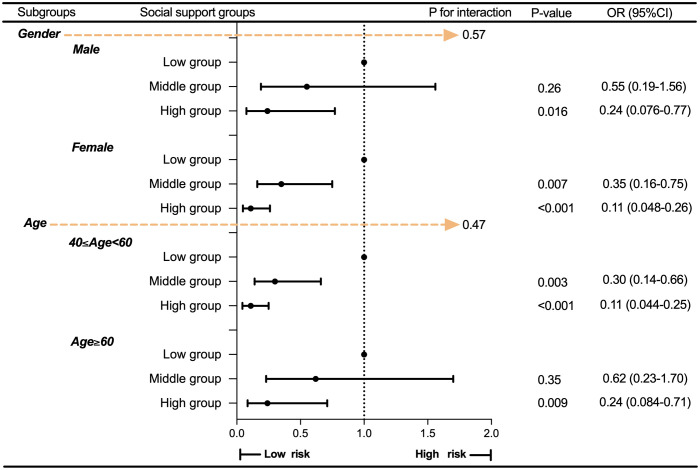
Subgroup analysis by social support groups. Abbreviations: MDD, Major Depressive Disorder; PSM, Propensity score matching; OR, odds ratio; CI, Confidence Interval.

**Fig 4:**
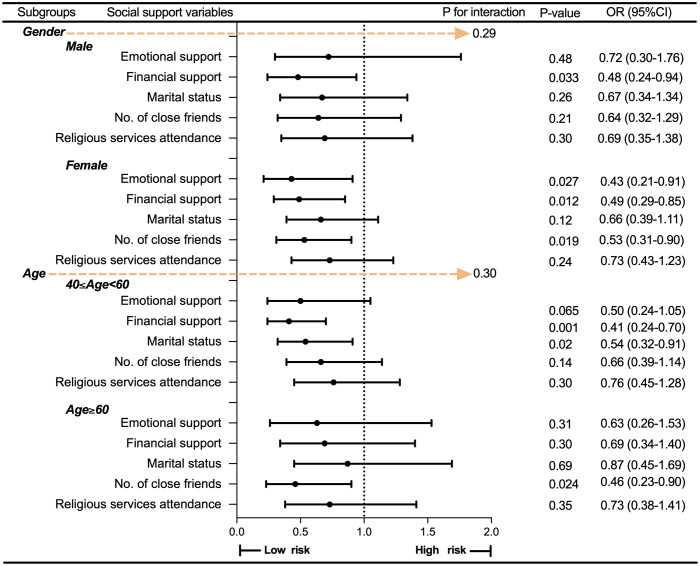
Subgroup analysis by all social support components. Abbreviations: MDD, Major Depressive Disorder; PSM, Propensity score matching; OR, odds ratio; CI, Confidence Interval.

Subgroup analyses were further performed to assess the robustness of the association between social support and MDD, with stratification by the two most common demographic variables: gender and age. As shown in S2–S3 Tables, Fig 3 and Fig 4, the association between social support and MDD remained robust regardless of how social support was categorized—whether by total score into three groups or by its five-dimensional components (all interaction P-values > 0.05).

Finally, sensitivity analyses further confirmed the robustness of our results. When social support was dichotomized by score into two groups (0–2 points and 3–5 points), the high social support group (3–5 points) had a significantly lower MDD incidence than the low social support group (0–2 points) (OR = 0.28, 95% CI: 0.19–0.42, P < 0.001; S4 Table).

As shown in the sensitivity analysis presented in S5 Table, when social support was categorized as a 6-level ordinal variable (scores: 0, 1, 2, 3, 4, 5), participants with social support scores of 3, 4, or 5 (higher levels of social support) had a significantly lower risk of MDD compared with the group with a social support score of 0 (the lowest level of social support). The specific results were as follows: Group with social support score of 5 vs. Group with social support score of 0 (OR = 0.12, 95% CI: 0.036–0.39, P < 0.001); Group with social support score of 4 vs. Group with social support score of 0 (OR = 0.063, 95% CI: 0.019–0.21, P < 0.001); and Group with social support score of 3 vs. Group with social support score of 0 (OR = 0.16, 95% CI: 0.051–0.48, P = 0.001).

## 4. Discussion

### 4.1. Main findings and strengths

This current study is the first research to determine the association between social support encompassing five dimensions and MDD identified using more rigorous diagnostic criteria (PHQ-9 ≥ 15 points) that are basically consistent with real-world epidemiological statistics in the general population of the United States aged 40 years and older. Recent epidemiological studies have shown that a PHQ-9 score of ≥10 significantly overestimates the prevalence of MDD. Therefore, in previous studies exploring the relationship between social support and MDD, the use of relatively lenient diagnostic criteria may have led to the inclusion of a large number of individuals who do not actually have MDD [20,21]. In the study of MH Chan et al., 10 (11.5%) participants were diagnosed with MDD using the criterion PHQ-9 ≥ 10, while only 2 (2.2%) were confirmed as MDD if the PHQ-9 ≥ 15 criterion was applied [20]. Many studies fail to distinguish between the severity levels of depression, which may introduce significant research bias. Since mild and moderate depression always account for the majority of all depression cases, while severe depression constitutes a minority, the final conclusions about the correlation between depression and social support may not be applicable to the minority group with more severe depressive symptoms [14,33,34]. Therefore, our study provides an important supplement to the current research field of social support and depression.

PHQ-9 ≥ 15 had a 95% specificity for MDD diagnosis and was considered to be more clinically relevant than PHQ-9 ≥ 10, by which we could exclude individual without MDD with a large extent [27]. On the other hand, though moderately severe and severe depression population (PHQ-9 ≥ 15) only had a relatively small percentage, their association with poor prognosis was quite strong [35–37]. Further clarification of the association between social support and MDD in this small percentage population is essential, which will help to maximize the use of limited medical resources in a targeted way.

After balancing the difference of baseline by PSM, the results of our analysis showed that participants with higher social support score had significantly lower risk of MDD, which was in accordance with the results of previous studies [13–15,20,33]. In the five dimensions of the social support scale, financial support was found to have the strongest correlation with MDD in our sample. Previous studies have found the similar results that higher levels of financial support, such as pension insurance and health insurance, was an important protective factor in the prevention and alleviation of depressive [19]. On the other hand, socio-economic adversity was believed to be associated with long-term non-recovery from depression in community settings [38].

Emotional support, having adequate close friends, and status of being married or living as married, also had a slightly weaker but statistically significant association with MDD than financial support in our study. Karen D et al. believed negative interaction and emotional support were risk and protective factor for depression, respectively [29]. Jensen et al. found that support from friends was much more strongly associated with depression than support from family [39]. Unmarried and living alone was proved significantly associated with the risk of MDD in previous study [15]. Compared to those who are unmarried or living alone, people who are married or living together may receive more invisible material and emotional support from their intimate spouse.

While religious support similarly had the weakest association with MDD. The relationships between religious belief as well as activity and depression were controversial in previous studies. Some believed religiosity played a protective role to against depression and frequent praying could reduce the distress level among patients with depression. Belief in God could reduce the stigma of using psychiatric services for depressed people [40]. Yee Chin Chai et al. found religious commitment was not significantly correlated with the depression [41]. Because religion is affected by regional and cultural differences, different time for religious activities and individual self-discipline are also confounding factors.

Our study found that social support has a certain ability to distinguish major depressive disorder (AUC = 0.67). Although the AUC does not reach a high discrimination level (above 0.8), this result still reflects the complexity of the pathogenesis of depression, which is jointly influenced by multiple factors such as genetics, environment, and psychology. As a cross-sectional study based on the general community population from NHANES, this research is the first to focus on the association between the five dimensions of social support and depression and conduct predictive modeling, providing basic data support for the construction of more accurate predictive models in future studies. In addition, in community mental health surveys, the level of social support can be used to quickly identify MDD high-risk groups, offering directions for early intervention (e.g., formulating targeted support strategies for groups with insufficient financial support).

Previous studies have shown that middle-aged and older populations tend to have reduced social support [42]. Our research could be beneficial for this high-risk group: providing them with more social support—particularly in terms of economic assistance and emotional care—or optimizing the use of social resources may help alleviate their economic burdens.

### 4.2. Limitations and future research direction

Our current study has several non-negligible limitations. First, we could not establish causal relationships between social support and MDD because our study is a cross-sectional study and therefore, reverse causality cannot be ruled out. Second, we have adjusted for key demographic variables, while residual confounding factors—such as socioeconomic status, detailed comorbidities, or the quality of social interactions—may still exist and potentially influence the results. Thirdly, while social support scales and the PHQ-9 questionnaire have been widely validated and applied, they may still be subject to recall and reporting biases, leading to potential bias. Additionally, the binary scoring method used for social support components may not fully capture the quality, intensity, or perceived adequacy of social support. Thirdly, due to sample size limitations, some subgroup analyses had wide CIs, so their results should be interpreted with caution. Finally, the results we obtained by using NHANES might not suitable for other populations or settings.

In the present study, we adopted depression diagnostic criteria that are more commonly used in clinical practice and more aligned with epidemiological principles, and identified a strong statistical association between social support and MDD. However, future validation in clinical practice—particularly among patients with chronic or critical illnesses—is warranted to optimize the allocation of social resources and thereby reduce healthcare burdens. Additionally, exploring the relationship between longitudinal changes in social support and depressive symptoms to establish a causal link is of critical importance. Although our study utilized 2007–2008 NHANES data and assessed social support across its five dimensions, the structure of social support may have evolved alongside societal development. In recent years, online social support (e.g., emotional connections via social media) has emerged as an important supplement to traditional offline support, a topic that also merits further investigation in future research.

## 5. Conclusions

Using more stringent criteria according with epidemiologic data for MDD, adequate social support, especially financial support, is associated with a lower risk of MDD in the general middle-aged and older population of the United States.

## Supporting information

S1 Table Description of the proportion of MDD in different social support groups after PSM. (XLSX)

S2 Table Age and gender subgroup analysis of MDD by social support groups after PSM. (XLSX)

S3 Table Age and gender subgroup analysis of MDD by all social support components after PSM. (XLSX)

S4 Table Sensitivity analysis of the correlation between social support groups (0–2 vs 3–5) and MDD after PSM. (XLSX)

S5 Table Sensitivity analysis of the correlation between social support groups (0 vs 1 vs 2 vs 3 vs 4 vs 5) and MDD after PSM. (XLSX)

S1 Fig Standardized mean differences before and after PSM. Abbreviations: PSM, Propensity score matching.
(TIF)

S2 Fig Area under the ROC curves indicate that social support score had a certain predictive value for MDD. Abbreviations: ROC, Receiver operating characteristic; AUC, Area under the curves; MDD, Major Depressive Disorder.
(PNG)

S1 File Graphical Abstract. (TIF)
