# Supplementary figures and images for: Lack of social support associated with major depressive disorder in middle-aged and older adults from the United States: A propensity score-matched cohort study
Source: PLoS One. 2026 Jan 16;21(1):e0340260. doi: 10.1371/journal.pone.0340260 (PMC12810787; doi:10.1371/journal.pone.0340260)

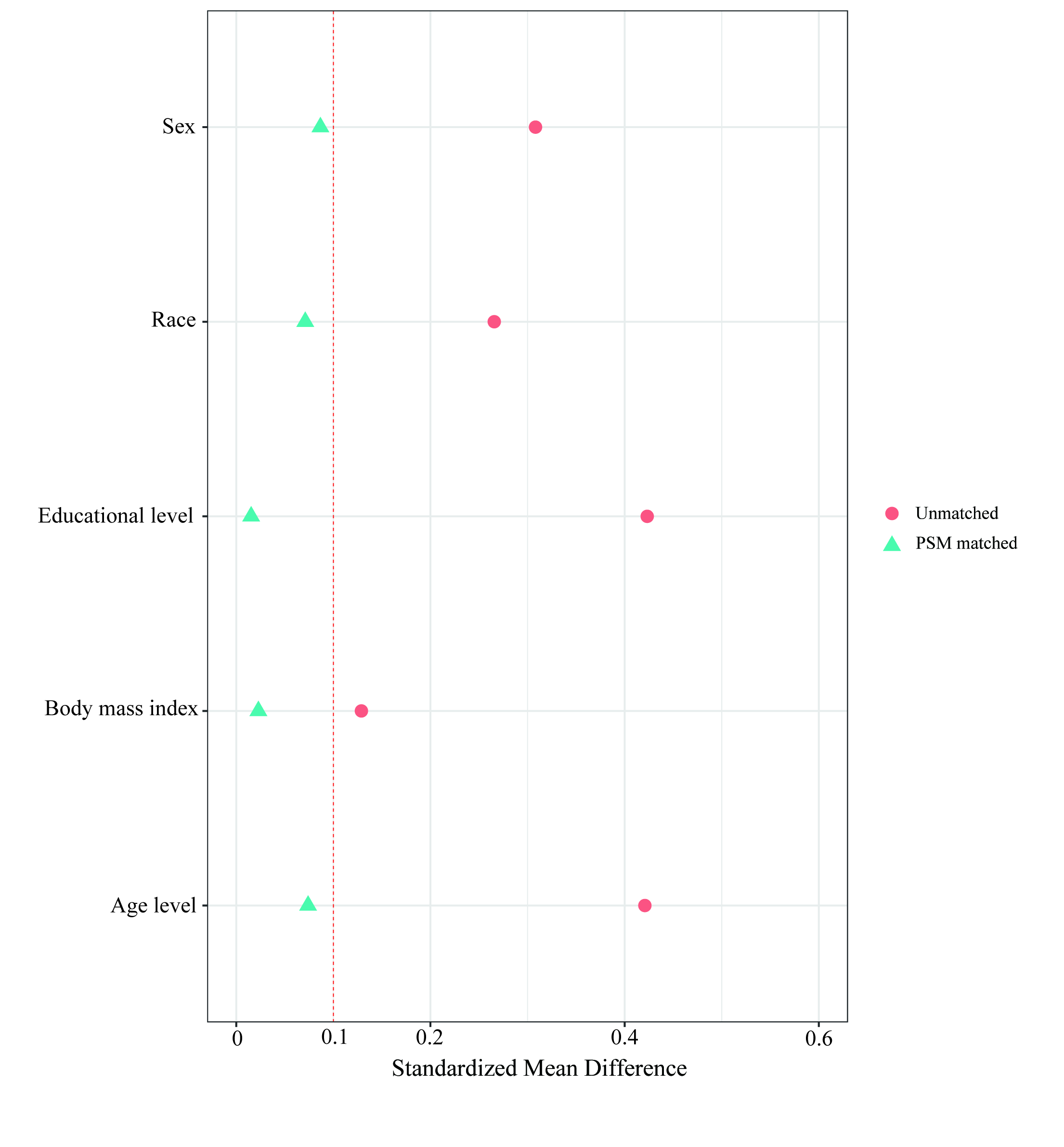

Supplement: S1 Fig — Abbreviations: PSM, Propensity score matching. (TIF) [file pone.0340260.s006.tif]

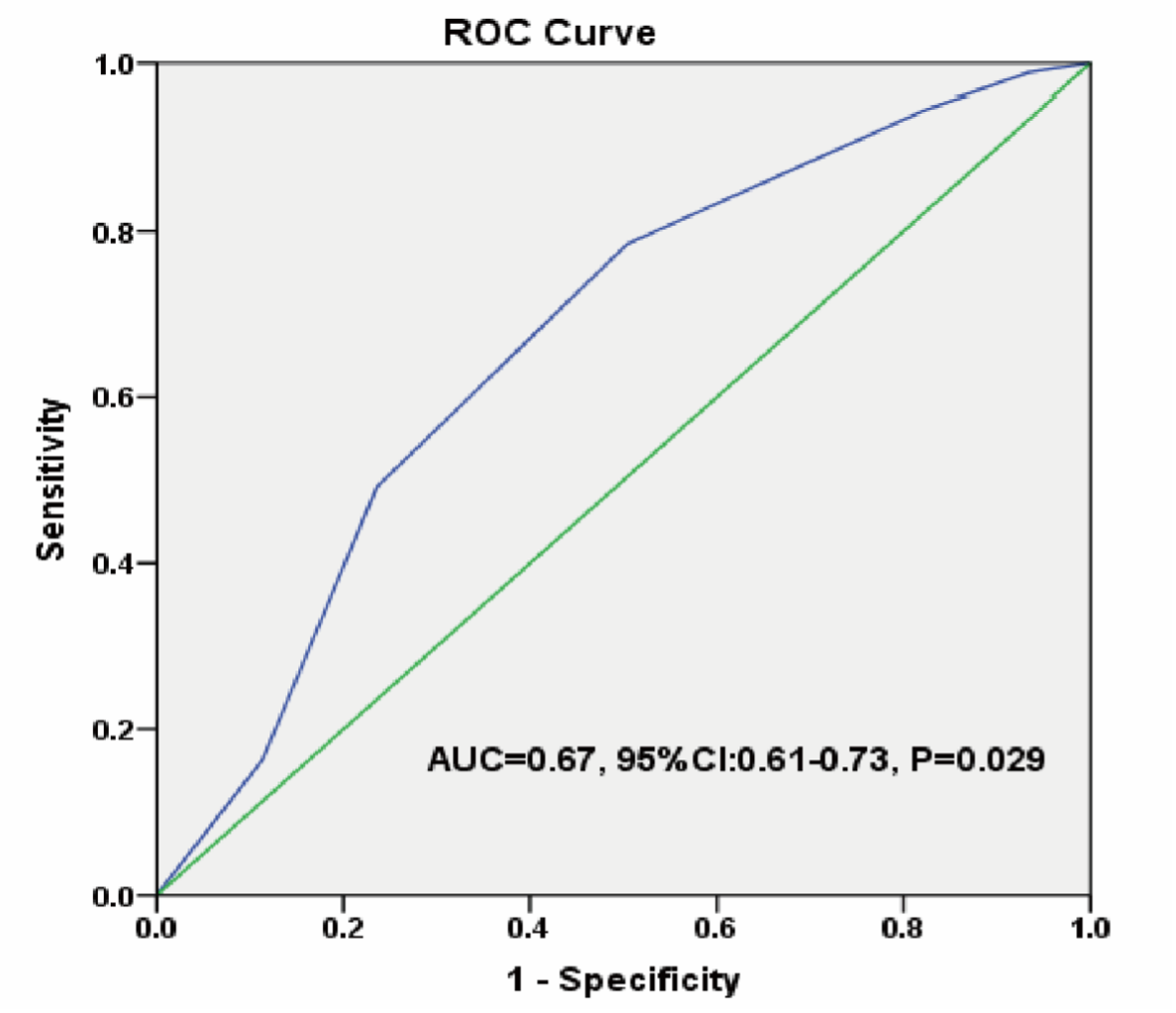

Supplement: S2 Fig — Abbreviations: ROC, Receiver operating characteristic; AUC, Area under the curves; MDD, Major Depressive Disorder. (PNG) [file pone.0340260.s007.png]

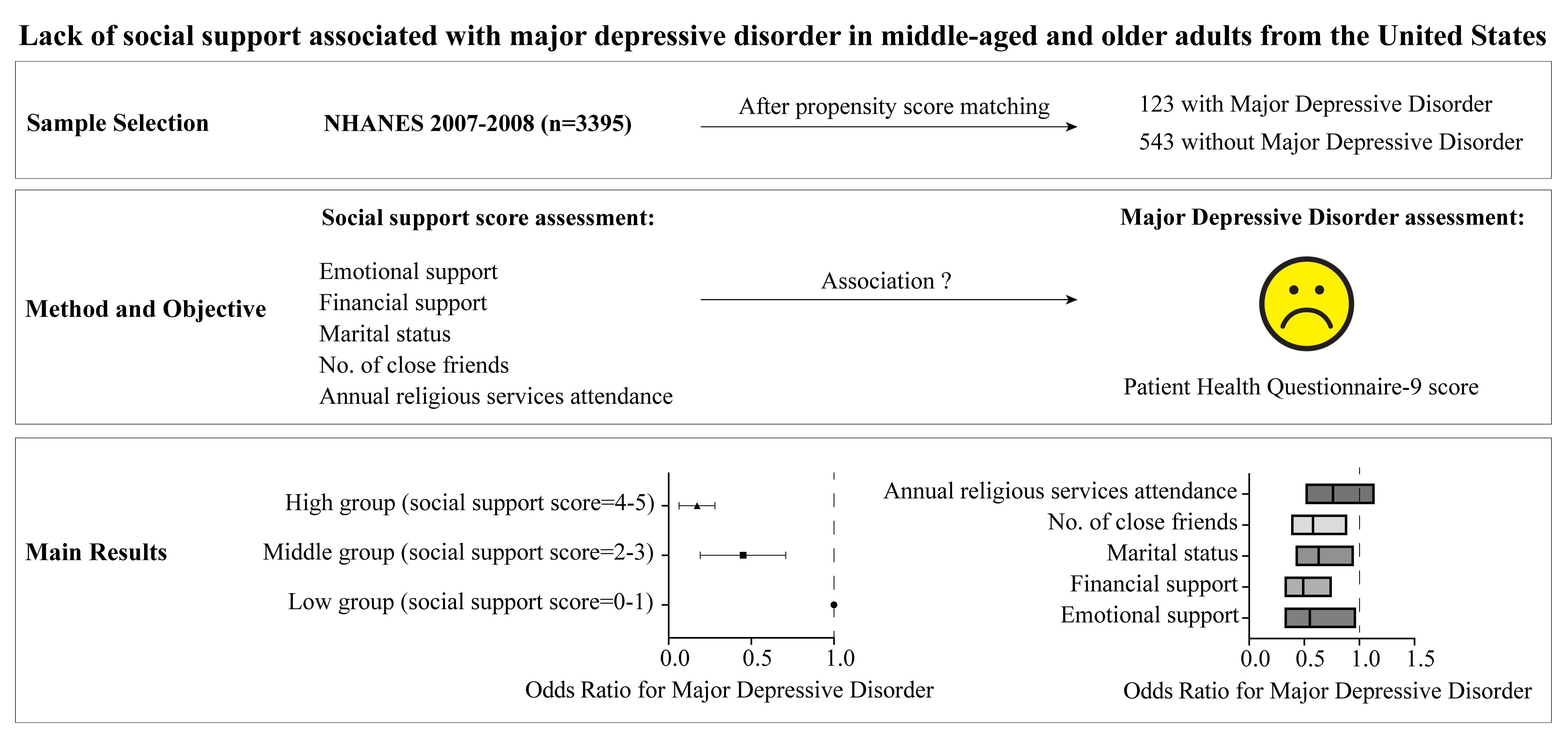

Supplement: S1 File — (TIF) [file pone.0340260.s008.tif]
